# Supplementary material for: Natriuretic Peptide Levels and Stages of Left Ventricular Dysfunction in Heart Failure with Preserved Ejection Fraction
Source: Biomedicines. 2023 Mar 13;11(3):867. doi: 10.3390/biomedicines11030867 (PMC10045594; doi:10.3390/biomedicines11030867)
Supplement: Supplementary file 1 [file biomedicines-11-00867-s001.zip › biomedicines-2275791-supplementary.pdf]

Supplementary Materials

Natriuretic Peptide Levels and Stages of Left Ventricular Dysfunction in Heart Failure with Preserved Ejection Fraction

Elisa Dal Canto, Marielle Scheffer, Kirsten Kortekaas, Annet Driessen-Waaijer, Walter J. Paulus and Loek van Heerebeek

Table S1. Sensitivity analysis comparing echocardiographic measures of HFpEF patients stratified by the NT-proBNP cut-off recommended for risk enrichment in HFpEF trials (=360 pg/ml).

Parameters	NT-proBNP < 360 pg/ml (n = 107)	NT-proBNP > 360 pg/ml (n= 45)	*P-value	†P-value
**LV structure and geometry**				
LVMI (g/m²)	83.9 ± 15.3	93.7 ± 20.9	0.019	0.034
PWTd (mm)	9.9 ± 1.5	10.1 ± 1.5	0.657	0.916
RWT	0.42 ± 0.07	0.42 ± 0.08	0.895	0.438
LVEDV (ml)	81.9 ± 19.6	78.6 ± 22.0	0.997	0.736
LVEDVI (ml/m²)	42.7 ± 9.6	42.1 ± 10.3	0.604	0.316
**LV systolic function**				
EF (%)	56.7 ± 5.0	55.4 ± 6.8	0.093	0.166
GLS (%)	19.1 ± 3.0	17.5 ± 2.2	0.008	0.115
**LV diastolic function**				
DT (ms)	209 ± 37	207 ± 51	0.405	0.447
Lateral E' (cm/s)	7.3 ± 1.6	7.4 ± 2.6	0.426	0.761
Septal E' (cm/s)	5.7 ± 1.2	5.7 ± 1.3	0.549	0.231
Mean E' (cm/s)	6.5 ± 1.2	6.5 ± 1.7	0.756	0.715
Mean A' (cm/s)	9.1 ± 1.8	7.3 ± 2.0	<0.001	0.006
Lateral E/E'	11.5 ± 3.5	15.0 ± 7.7	0.003	0.006
Septal E/E'	14.7 ± 4.3	18.4 ± 6.2	0.018	0.139
Mean E/E'	12.8 ± 3.6	16.0 ± 6.1	0.010	0.033
**LA structure and function**				
Max LAVI (ml/m²)	40.4 ± 9.7	50.7 ± 13.1	<0.001	<0.001
Pre-A LAVI (ml/m²)	30.5 ± 8.1	38.4 ± 12.4	<0.001	0.001
Min LAVI (ml/m²)	22.3 ± 6.8	31.8 ± 11.1	<0.001	<0.001
LA global ef (%)	46.7 ± 8.0	39.6 ± 9.2	0.001	0.013
LA passive ef (%)	25.3 ± 7.5	25.3 ± 8.2	0.902	0.410
LA active ef (%)	28.1 ± 9.1	21.4 ± 8.3	0.015	0.071
LA compliance	3.5 ± 1.1	2.5 ± 1.0	<0.001	0.0102
**RV and RA structure and function**				
TAPSE (mm)	22.7 ± 3.4	21.0 ± 3.7	0.074	0.381
RV FAC (%)	42.9 ± 11.0	39.3 ± 9.6	0.066	0.072
RV strain (%)	24.5 ± 6.5	18.5 ± 6.8	0.037	0.513
TR velocity (m/s)	2.58 ± 0.45	2.74 ± 0.27	0.427	0.745
Max RAV (ml)	40.2 ± 11.8	50.6 ± 19.9	0.001	0.032

Data are shown as n (%) or mean ± SD or median (interquartile range). LV: left ventricular. LVMI: LV Mass Index. PWTd: Posterior Wall Thickness in diastole. RWT: Relative Wall Thickness. EDV: End-diastolic Volume. EDVI: EDV Index. EF: Ejection Fraction. SV: stroke volume. SVI: SV index. GLS: Global Longitudinal Strain. DT: Deceleration Time. E′: peak early diastolic tissue velocity. A′ mean: peak late diastolic tissue velocity. E/E′: peak early filling over early diastolic tissue velocities ratio. LA: left atrial. LAVI max, pre-A, min: LA Volume Index maximal, at the onset of A wave, minimal. ef: emptying fraction. RV: right ventricular. RA: right atrial. TAPSE: Tricuspid Annular Plane Systolic Excursion. FAC: fractional area change. RAV: RA volume. TR: Tricuspid Regurgitation. The P-value refers to the comparison between low and high NT-proBNP groups. *The

comparison is adjusted for age, gender, BMI and creatinine. † The comparison is additionally adjusted for systolic blood pressure, use of loop and thiazide diuretics and beta-blockers.

Table S2. Clinical and echocardiographic characteristics of the overall study population and of the subgroup undergoing CMR measurements.

	**Overall population (n=152)**	**MRI population (n=72)**	***P-value**
Age (yr)	70.9 ± 8.9	71 ± 8	0.945†
Sex (% women)	144 (92.7)	67 (93.1)	0.941†
BMI (kg/m2)	31.7 ± 6.4	30.6 ± 5.3	0.312†
Hypertension n (%)	109 (71.7)	47 (65.2)	0.397
T2DM n (%)	52 (34.2)	21 (29.2)	0.718
Loop diuretics use n (%)	55 (36.2)	21 (29.2)	0.367
eGFR (mL/min/1.73m^2)	65.8 ± 18.8	68.5 ± 15.6	0.363†
NT-proBNP (pg/mL)	194.9 (84.7-436.4)	169.5 (84.6-364.4)	0.406
**Echocardiographic measures**			
LVMI (g/m^2)	87.0 ± 17.6	82.7 ± 16.8	0.134
LVEF (%)	56.2 ± 5.6	57.5 ± 6.0	0.204
Mean E’ (cm/s)	6.5 ± 1.4	6.6 ± 1.5	0.828
Mean E/E’	13.9 ± 4.7	13.4 ± 4.7	0.670
Max LAVI (ml/m^2)	43.6 ± 11.8	43.4 ± 12.4	0.891
La global ef (%)	44.6 ± 9.0	44.5 ± 9.9	0.876
TAPSE (mm)	22.1 ± 3.5	22.1 ± 3.6	0.638
TR velocity (m/s)	2.65 ± 0.40	2.66 ± 0.43	0.792
Max RAV (ml)	43.2 ± 15.1	42.6 ± 17.8	0.968

Data are shown as n (%) or mean ± SD or median (interquartile range). T2DM: Type 2 Diabetes Mellitus. eGFR: estimated Glomerular Filtration Rate. NT-proBNP: N-terminal pro Brain Natriuretic Peptide. LV: left ventricular. LVMI: LV Mass Index. EF: Ejection Fraction. E′: peak early diastolic tissue velocity. E/E′: peak early filling over early diastolic tissue velocities ratio. LA: left atrial. Max LAVI: LA Volume Index maximal. TAPSE: Tricuspid Annular Plane Systolic Excursion. TR: Tricuspid Regurgitation. RAV: Right atrial volume. *The P-value refers to the comparison between the whole population and the subgroup with MRI measurements and the analysis is adjusted for age, gender, BMI and creatinine. † The crude value is provided. In this subgroup, increasing values of log-transformed-NT-proBNP were significantly associated with a greater proportion of ECV (β=1.82, 95% CI: 0.19;3.44, P=0.029 in the fully adjusted model, Table 4), whereas no significant associations were observed with myocardial T1 pre-contrast and post-contrast times.
